# Expression and clinical implications of PARs in the stenotic tissue of ureteropelvic junction obstruction

**DOI:** 10.3389/fped.2023.1286786

**Published:** 2023-12-15

**Authors:** Tianyi Wang, Mingcui Fu, Xiangming Yan, Hongcheng Song, Weiping Zhang

**Affiliations:** ^1^ Department of Urology, Children’s Hospital of Soochow University, Suzhou, Jiangsu, China; ^2^ Department of Urology, Beijing Children’s Hospital, National Center for Children's Health, Capital Medical University, Beijing, China

**Keywords:** ureteropelvic junction obstruction, protease activated receptor, SIP syncytium, hydronephrosis, children

## Abstract

**Objective:**

To explore the expression and clinical implications of protease activated receptors (PARs) in the pathogenesis of children with ureteropelvic junction obstruction (UPJO).

**Material and methods:**

Immunohistochemistry was employed to investigate the distribution of PARs in both normal human ureteropelvic junction (UPJ) and cases of UPJO. Furthermore, PAR gene expression levels were assessed using real-time PCR (RT-PCR), and the patients in the UPJO group were stratified according to the Onen grading system. Subsequently, the clinical implications of PARs in UPJO were explored through RT-PCR analysis.

**Results:**

Immunofluorescence showed robust PAR2 expression in the control group compared with the UPJO group. The results of RT-PCR analysis revealed a significant decrease in the relative mRNA expression of PAR2 in the UPJO group compared to the control group. Notably, the relative RNA expression of PAR1 was significantly lower in the Onen-4 group compared to the control group. Furthermore, the relative mRNA expression of PAR2 exhibited a statistically significant difference among the Onen-3 group, Onen-4 group, and control group.

**Conclusions:**

PARs are widely distributed throughout the SIP syncytium of the UPJ and play a role in maintaining smooth muscle cells (SMCs) membrane potential by interacting with interstitial cells of Cajal (ICCs), as well as platelet-derived growth factor receptor alpha-positive cells (PDGFR α+ cells). The decreased expression of PAR1 suggests a higher preoperative Onen grade in UPJO patients. Furthermore, the downregulation of PAR2 effects at the UPJ may be involved in the loss of inhibitory neuromuscular transmission, disrupting the rhythmic peristalsis of the UPJ.

## Introduction

Ureteropelvic junction obstruction (UPJO) is regarded as a functionally predominant impairment of urinary transport from the renal pelvis to the ureter (1). It occurs in 1 per 1,000 to 2,000 live births with a 3:1 male-to-female ratio and a 2:1 left-to-right ratio approximately (2, 3). However, the reasons for this discrepancy remain unclear. The kidney is a “silent” organ in the body and most children with UPJO exhibit no obvious symptoms in the early stages, which results in delayed diagnosis and treatment and may lead to impaired kidney function in the long term. Numerous pathophysiological mechanisms have been hypothesized, but remain controversial.

The present research body provides evidence that the pathogenesis of UPJO at the ureteropelvic junction (UPJ) is linked to abnormal innervation, dysfunction of smooth muscle cells accompanied by collagen replacement, and abnormal expression of electroactive cells (2, 4, 5). Among these, a growing number of electroactive cell research studies have been carried out in recent years. Previous studies have demonstrated the presence of two distinct types of interstitial cells in the human UPJ that can conduct electrical activity to smooth muscle cells (SMCs), namely interstitial cells of Cajal (ICCs) and platelet-derived growth factor receptor alpha-positive cells (PDGFR α+ cells) (6, 7). ICCs and PDGFR α+ cells are electrically coupled to smooth muscle cells by gap junctions, and the three together form a functional SIP (SMCs-ICCs-PDGFRα positive cells) syncytium that expresses a wide range of ion channels and receptors. They are involved in neurotransmission and neuromodulation, which in turn modulates SMCs contraction (7, 8). Moreover, studies in the gastrointestinal tract of mice and humans revealed that protein kinase-activated receptors (PARs) are widely distributed in the SIP syncytium and are involved in the regulation of ion transport, inflammatory responses, and other physiological and pathological processes (9–12).

Currently, no study has reported the distribution and expression changes of PARs in the human UPJ, and the role of PARs in the pathogenesis of UPJO remains unknown. This study focused on the expression and distribution of PARs in SIP syncytium in the human UPJ and explored their effects and clinical implications in the pathogenesis of children with congenital hydronephrosis.

## Materials and methods

### Patients and samples

This prospective study was approved by the Medical Ethics Committee of Beijing Children's Hospital, Capital Medical University, and informed consent was obtained from the guardians of all the enrolled patients. The UPJO specimen repository was established between January 2020 and December 2021. UPJ samples were obtained from 16 patients (mean age 18.1 months, range 3–41 months) undergoing pyeloplasty for intrinsic UPJO. Renograms and ultrasound scans were performed to diagnose UPJO. Only cases of intrinsic obstruction that were confirmed intraoperatively were included in our study. Pyeloplasty was indicated in the setting of any of the following: a differential renal function (DFR) <40% or a fall of >10% on serial 99mTc-DTPA diuretic renography; worsening hydronephrosis with a renal pelvis anteroposterior diameter of >3 cm on ultrasound scan; symptomatic children. Data were collected from all patients, including gender, affected side, age at surgery, surgical procedure, anteroposterior diameter of the renal pelvis (APD), Society for Fetal Urology (SFU) grading of hydronephrosis, Onen grading of hydronephrosis, and differential renal function (DRF). In the control group, 8 patients (mean age 34 months, range 11 months–73 months) underwent nephrectomy for nephroblastoma. All patients in the control group had no clinical or radiographic evidence of UPJO and their UPJ specimens were free of tumor cell invasion.

In each case, a 5–6 mm region of the UPJ was taken for the analysis. Each sample was longitudinally resected and divided into two segments. One segment was promptly preserved in 4% formalin with phosphate buffer and embedded in paraffin for immunofluorescence staining and confocal microscopy, while the other segment was rapidly frozen in liquid nitrogen and stored at −80°C for RNA extraction. Notably, this study excluded the insufficient UPJ samples.

### Immunofluorescence staining and confocal microscopy

To assess the distribution and expression of PARs in the SIP syncytium, the following double-labeling was performed in the UPJO and control samples.

(1) PAR1 and c-kit (a marker of ICCs) immunofluorescence double staining.
(2) PAR1 and phalloidin (a marker of SMCs) double staining.
(3) PAR1 and PDGFRα (a marker of PDGFRα+ cells) double staining.
(4) PAR2 and c-kit double staining.
(5) PAR2 and phalloidin double staining.
(6) PAR2 and PDGFRα double staining.

Paraffin-embedded blocks of 16 UPJ obstruction and 8 UPJ control samples were sectioned transversely at a thickness of 10 μm. All slides were fixed with 10% buffered formalin for 5 min. The sections were then permeabilized with 0.3% Triton X-100 for 10 min at room temperature. Following a 30-min blocking step using 10% bovine serum albumin (Solarbio, Beijing, China), the sections were incubated overnight at 4°C with a mixture of primary antibodies diluted in phosphate-buffered saline containing 1% bovine serum albumin. The following primary antibodies were used: rabbit anti-PAR1 (1:200, AF0263, Affinity); rabbit anti- PAR2 (1: 100, AB180953, Abcam); Mouse anti- C-kit (1: 50, SC-13508, Santa); Mouse anti- PDGFR α (1: 50, SC-398206, Santa); Phalloidin (1: 100, ab150080, Abcam). Subsequently, the sections were rinsed in phosphate-buffered saline+ ddH2O and incubated with the corresponding secondary antibodies (HRP-labeled goat anti-rabbit IgG, 1:200, 074-1506, Abcam) for a duration of 2 h at ambient temperature. To visualize the nuclei, the samples were counterstained with 4′6-diamidino-2-phenylindole (DAPI) for a period of 10 min. Finally, all sections were washed, mounted, and coverslipped. The sections were evaluated independently by two investigators using confocal microscopes (Nikon Ci-S, Japan) and the Vectra Polaris automated quantitative pathology imaging system (PerkinElmer).

### RNA extraction and quantitative polymerase chain reaction

TRIZOL reagent was used to extract total RNA, and the cDNA Synthesis Kit from Yeasen Biotech was used to synthesize first-strand cDNA, following the manufacturer's instructions. RT-PCR was performed using the SYBR FAST qPCR Kit Master Mix (2×) Universal (Kapa Biosystems) on the ABI 7500 Real-time PCR Instrument (Life Technologies, CA, USA) in a 20 μl system. In addition, the SYBR FAST qPCR Kit Master Mix (2×) Universal (Kapa Biosystems) was utilized to perform RT-PCR on the ABI 7500 Real-time PCR Instrument (Life Technologies, CA, USA) in a 20 μl system. A total of 6 control samples and 8 UPJ samples were employed for RT-PCR. The following PCR primers were used: PAR1fw: 5′- TGCCTACTTTGCCTACCTCC -3′, PAR1rv: 5′- GTAGACGTACCTCTGGCAC -3′, PAR2fw: 5′- GCGATCTTCTGCCATGGATG -3′, PAR2rv: 5′- AGATCAGGTACATGGCCAGG -3′, GAPDHfw: 5′- GGAGTCAACGGATTTGGT -3′, GAPDHrv: 5′- GTGATGGGATTTCCATTGAT -3′. The relative changes in the expression levels of PAR1 and PAR2 were normalized against the levels of GAPDH gene expression in each sample using the ΔΔCt method. Each sample and primer were subjected to triplicate experiments. Furthermore, the relative mRNA expression levels of PAR1 and PAR2 in different Onen grades of preoperative hydronephrosis were investigated to explore their clinical implications.

### Statistical analysis

Statistical analysis was conducted using the SPSS 26.0 statistical software (IBM Corporation, New York, United States). The K-S single sample test was used to determine whether the data were normally distributed. The numerical data were then presented as mean ± standard deviation (SD). Data not conforming to a normal distribution were expressed as medians and quartiles. The differences between two groups were analyzed using either the Student's *t*-test or the Mann–Whitney *U*-test, depending on the distribution of the data. For comparisons among three groups, one-way ANOVA and the Kruskal–Wallis test were employed. In this study, *P* < 0.05 was considered statistically significant.

## Results

### Confocal immunofluorescence

**Figure 1:**
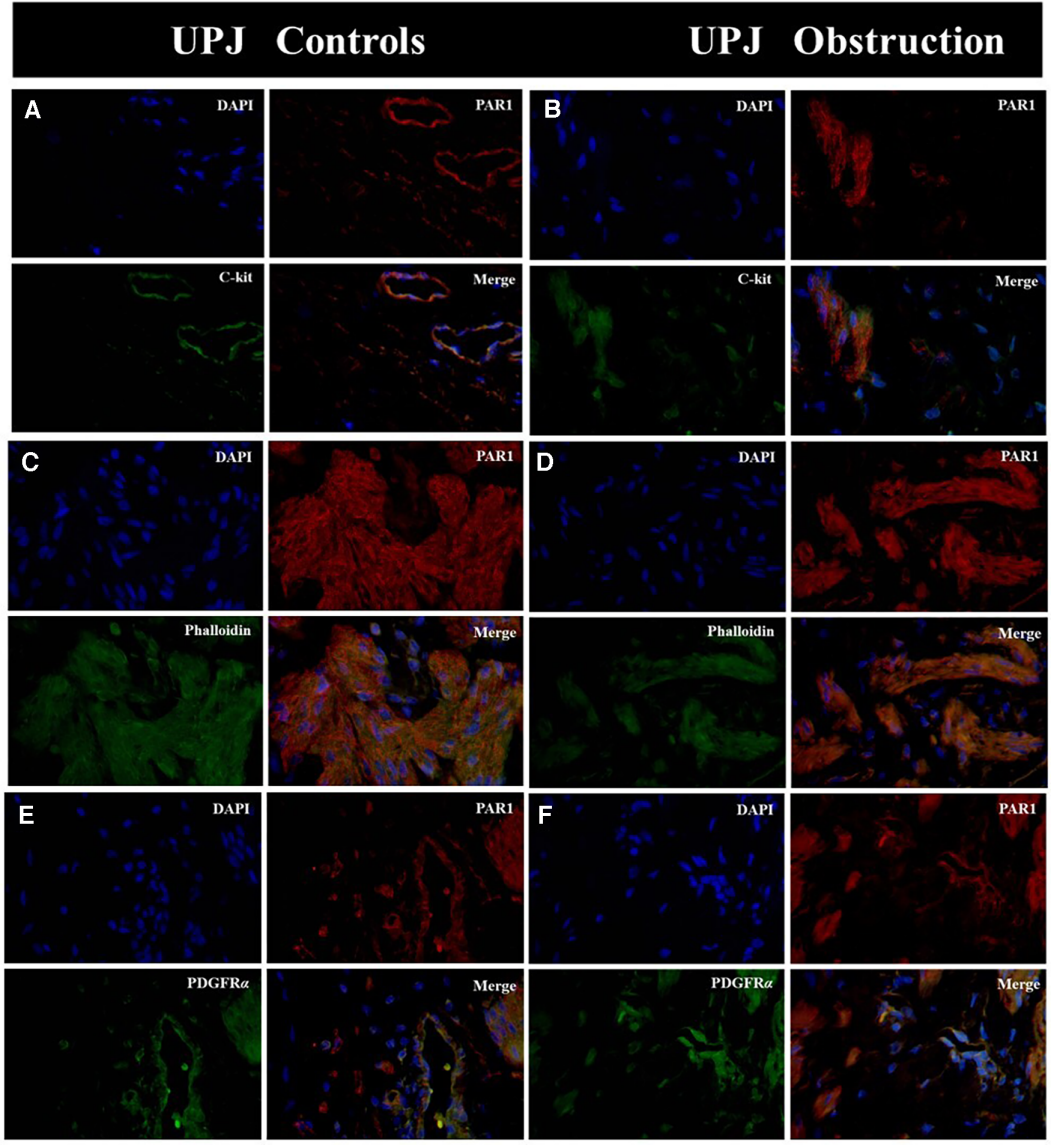
Immunofluorescence double staining was conducted on PAR1 with various types of SIP syncytium. (**A**) Immunofluorescence double staining of PAR1 (red) and c-kit (green) in UPJ control; (**B**) immunofluorescence double staining of PAR1 (red) and c-kit (green) in UPJO; (**C**) immunofluorescence double staining of PAR1 (red) and phalloidin (green) in UPJ control; (**D**) immunofluorescence double staining of PAR1 (red) and phalloidin (green) in UPJO; (**E**) immunofluorescence double staining of PAR1 (red) and PDGFRα (green) in UPJ control; (**F**) immunofluorescence double staining of PAR1 (red) and PDGFRα (green) in UPJO. The blue areas in all images represent DAPI-labelled nuclei. Nuclei were counterstained with DAPI (blue) in all panels. Bar = 50 μm in all panels.

Immunofluorescence double-staining microscopy analysis revealed that PAR1 was co-expressed with ICCs, SMCs, and PDGFRα+ cells in both the UPJO and control groups. PAR1 was extensively distributed in the cytomembranes of all cell types within the SIP syncytium, showing no difference in PAR1 expression between the UPJO and control groups (Figure 1).

**Figure 2:**
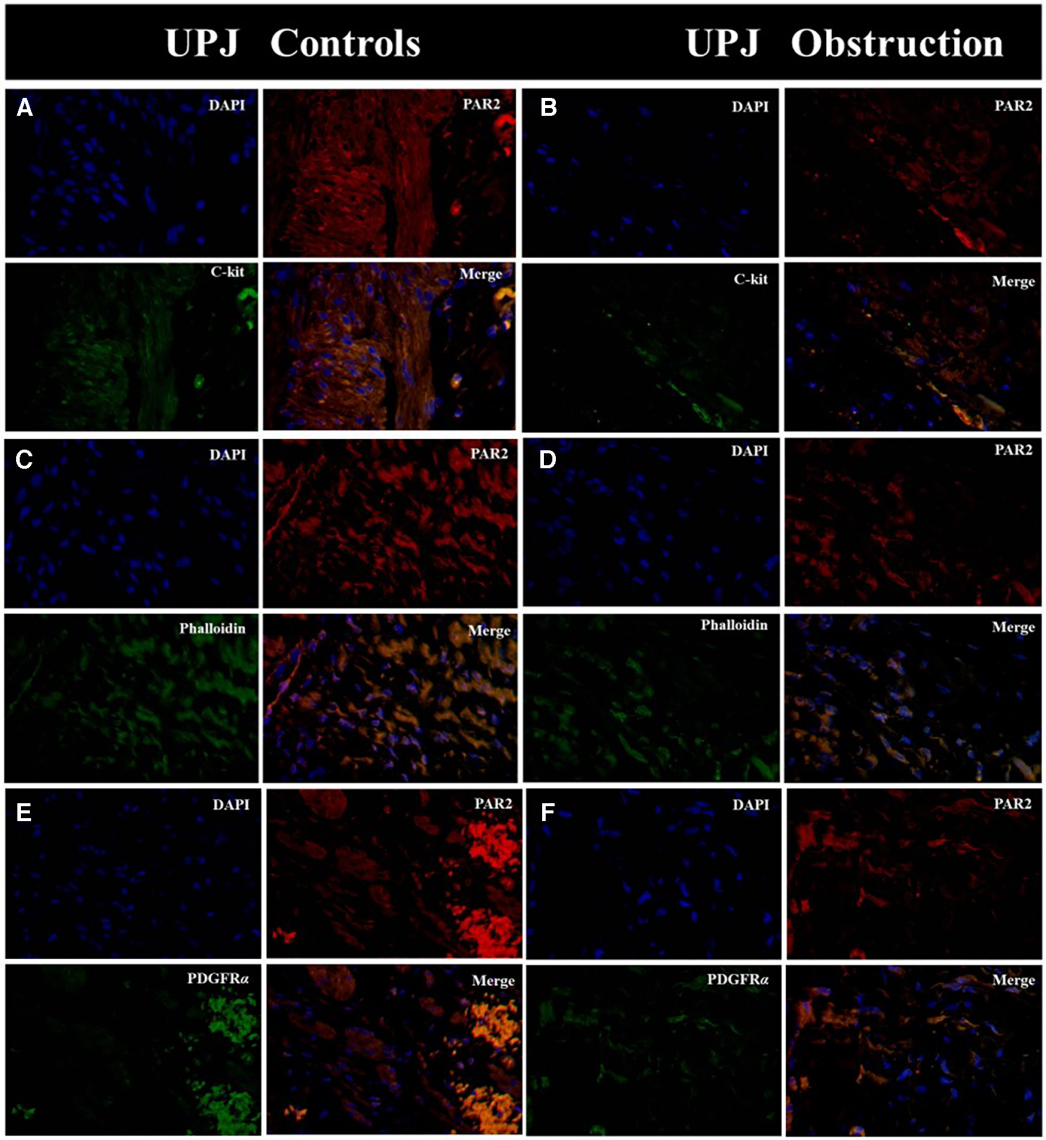
Immunofluorescence double staining was conducted on PAR2 with various types of SIP syncytium. (**A**) Immunofluorescence double staining of PAR2 (red) and c-kit (green) in UPJ control; (**B**) immunofluorescence double staining of PAR2 (red) and c-kit (green) in UPJO; (**C**) immunofluorescence double staining of PAR2 (red) and phalloidin (green) in UPJ control; (**D**) immunofluorescence double staining of PAR2 (red) and phalloidin (green) in UPJO; (**E**) immunofluorescence double staining of PAR2 (red) and PDGFRα (green) in UPJ control; (**F**) immunofluorescence double staining of PAR2 (red) and PDGFRα (green) in UPJO. The blue areas in all images indicate DAPI-labelled nuclei. Nuclei were counterstained with DAPI (blue) in all panels. Bar = 50 μm in all panels.

Immunofluorescence double staining microscopy revealed that PAR2 was co-expressed with ICCs, SMCs, and PDGFRα+ cells in both the UPJO and control groups. Additionally, PAR2 was extensively distributed in the cytomembranes of all cell types within the SIP syncytium. Nevertheless, a lower PAR2 expression was observed in the UPJO group compared to the control group (Figure 2).

### Relative mRNA expression levels

**Figure 3:**
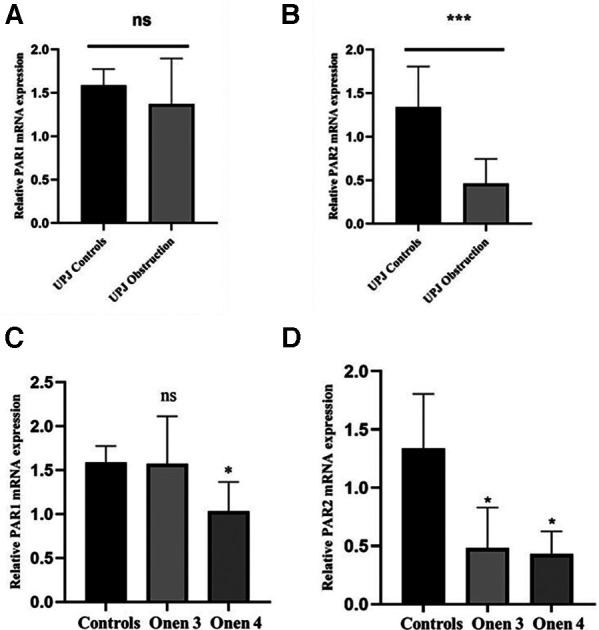
(**A**) the relative mRNA expression levels of PAR1 between the UPJ obstruction group (*n* = 8) and the control group (*n* = 6). (**B**) The relative mRNA expression levels of PAR2 between the UPJ obstruction group (*n* = 8) and the control group (*n* = 6). (**C**) The relative mRNA expression levels of PAR1 between the UPJ control group (*n* = 6), the Onen-3 group (*n* = 5), and the Onen-4 group (*n* = 3). (**D**) The relative mRNA expression levels of PAR2 between the UPJ control group (*n* = 6), the Onen-3 group (*n* = 5), and the Onen-4 group (*n* = 3).

In order to corroborate the findings obtained through immunofluorescence microscopy, reverse transcription polymerase chain reaction (RT-PCR) was performed to examine the relative expression levels of PAR1 and PAR2 mRNA. The RT-PCR analysis confirmed the presence of mRNA transcripts for all targets. Notably, there was no statistically significant difference in the relative expression levels of PAR1 mRNA between the UPJO group and the control group (Figure 3). The relative mRNA expression levels of PAR1 in the UPJO group (*n* = 8) and control group (*n* = 6) were 1.37 ± 0.53 and 1.59 ± 0.19, respectively (*P *= 0.3053 > 0.05). Specifically, the relative mRNA expression levels of PAR2 in the UPJO group (*n* = 8) and control group (*n* = 6) were determined to be 0.46 ± 0.28 and 1.34 ± 0.47, respectively (*P *= 0.0009 < 0.05), revealing a significantly lower relative mRNA expression levels of PAR2 in the UPJO group compared with the control group (Figure 3).

### Expression of PARs in different Onen grades

In this study, all 8 patients in the UPJO group had a preoperative SFU grading of 4. Furthermore, the patients in the UPJO group were further categorized based on the Onen grading system (13). This system differentiates SFU grade 4 into Onen grade 3 and Onen grade 4. Among the 8 cases in the UPJO group, 5 cases were categorized as Onen-3 grade and 3 cases as Onen-4 grade. The participants were subdivided into the Onen grade 3 group (*n* = 5), the Onen grade 4 group (*n* = 3), and the control group (*n* = 6) for the PAR1 and PAR2 relative mRNA expression level analyses.

The mRNA expression levels of PAR1 were found to be 1.59 ± 0.19, 1.57 ± 0.54, and 1.03 ± 0.33 in the control, Onen grade 3, and Onen grade 4 groups, respectively (Figure 3). Statistical analysis revealed no significant difference in PAR1 mRNA expression among the three groups (*P *= 0.06 > 0.05). However, a significantly lower PAR1 mRNA expression was observed in the Onen grade 4 group compared to the controls (*P* = 0.036 < 0.05). In contrast, no significant difference in the relative mRNA expression of PAR1 was found between the Onen grade 3 group and the control group (*P *= 0.65 > 0.05). Similarly, there was no significant difference in the relative mRNA expression of PAR1 between the Onen grade 4 group and the Onen grade 3 group (*P *= 0.15 > 0.05).

The mRNA expression levels of PAR2 were quantified as 1.34 ± 0.47, 0.48 ± 0.34, and 0.43 ± 0.20 in the control, Onen grade 3, and Onen grade 4 groups, respectively, as displayed in Figure 3. The statistical analysis revealed a significant difference in mRNA expression levels of PAR2 among the three groups (*P *= 0.005 < 0.05). Specifically, the relative mRNA expression of PAR2 was significantly lower in the Onen grade 4 group compared to the control group (*P *= 0.02 < 0.05). Similarly, the relative mRNA expression of PAR2 in the Onen grade 3 group was significantly lower than the control group (*P *= 0.01 < 0.05). Nevertheless, no significant difference in relative mRNA expression of PAR2 was observed between the Onen Grade 4 group and the Onen Grade 3 group (*P *= 0.98 > 0.05).

## Discussion

The most prevalent cause of obstruction at the ureteropelvic junction (UPJ) is intrinsic obstruction caused by an adynamic stenotic segment, resulting in an incomplete functional obstruction due to peristalsis failure (2). This study specifically examined cases of intrinsic UPJ obstruction. Various pathophysiological mechanisms have been hypothesized to explain its etiology; however, these mechanisms do not account for the gender and lateral distribution bias observed in UPJ obstruction patients and provide no guidance on the optimal timing for surgical intervention. Furthermore, recent research has indicated the presence of diverse cellular entities implicated in the pathological mechanisms of UPJO.

Notably, researchers have focused their efforts on electroactive cells. The current studies have revealed that the interstitial cells of Cajal (ICCs) exhibit a parallel arrangement with the inner longitudinal muscle fibers and are significantly associated with the distribution of nerve fibers. This observation suggests the involvement of ICCs in neural signaling and the regulation of ureteral peristalsis (6, 14). The activation of anoctamin-1 (ANO1) on the cell membranes of ICCs initiates the generation of superimposed ANO1 channel currents, resulting in the propagation of a slow wave throughout the ICCs network (15, 16). This wave simultaneously depolarizes the SMCs through gap junctions and activates L-type calcium channels on the SMC membranes, ultimately leading to smooth muscle contraction (15). Additionally, recent research has identified a different type of interstitial cell, PDGFR α^+^ cells, in the human UPJ, which may also be involved in the development of UPJO (7). PDGFR α^+^ cells express large quantities of small conductance calcium-activated potassium channel 3 (SK3), which is entirely reliant on heightened intracellular calcium ions (7, 17). Subsequent activation of this channel leads to an efflux of intracellular potassium ions, resulting in hyperpolarization of the membrane potential of PDGFR α^+^ cells. This hyperpolarization is facilitated by gap junctions with SMCs and inhibits L-type calcium channel currents in the SMC cell membrane, ultimately inducing smooth muscle relaxation (18).

Our previous study confirmed the proximal location of PDGFR α^+^ cells to ICCs, SMCs, and nerve cells. This finding indicates that SMCs, ICCs, and PDGFRα^+^ cells operate collectively as the SIP syncytium in the human ureteropelvic junction (17). The contractile behavior of SMCs in the SIP syncytium relies on the regulation of ICCs and PDGFRα^+^ cells, suggesting their relatively passive nature. In order to enhance our understanding of the causes underlying UPJO, the upstream regulatory factors influencing the SK3 channels were investigated. PARs are a class of G protein-coupled receptors characterized by their seven transmembrane segments (9, 12). Among the PAR isoforms, namely PAR1, PAR2, PAR3, and PAR4, PAR3 and PAR4 are predominantly expressed on platelet surfaces and play a crucial role in the context of thrombin response (19). Conversely, PAR1 and PAR2 are ubiquitously expressed across various tissues and cells and are involved in the regulation of ion transport as well as the modulation of smooth muscle cell contraction (11, 12). Sung et al. (19) conducted a study that revealed the presence of PAR1 and PAR2 in the cell membranes of various types of SIP syncytium. The expression levels of these receptors were found to be significantly higher in PDGFRα^+^ cells than in ICCs and SMCs. Moreover, patch clamp experiments revealed that the activation of these receptors participated in the regulation of smooth muscle contraction and relaxation (19). The immunofluorescence staining results of our study were in accordance with the aforementioned research, indicating that PAR1 and PAR2 were co-expressed with ICCs, SMCs, and PDGFRα^+^ cells in both the UPJO and control cohorts. PAR1 and PAR2 were both widely distributed across the cytomembranes of all cell types within the SIP syncytium. In the field of gastrointestinal research, the activation of PARs leads to a temporary rise in intracellular calcium levels within ICCs (12). This increase in calcium impacts slow wave currents and subsequently affects the excitability of smooth muscle. Additionally, PAR activation can induce the release of calcium from the endoplasmic reticulum of PDGFRα^+^ cells. This released calcium then activates SK3 channels and inhibits the contraction of adjacent smooth muscle cells (11). Consistent with the observations made by Tomuschat et al. (11), our immunohistochemistry investigation verified a reduction in PAR2 expression within the UPJO group compared to the control group. Moreover, the relative mRNA expression levels of PAR2 were significantly lower in the UPJO group compared with the control group. PARs exhibit differential expression in various cell types within the SIP syncytium and maintain the equilibrium between the effects of ICC-ANO1 and PDGFRα^+^-SK3 on smooth muscle, thereby regulating rhythmic contraction at the UPJ. In summary, within the stenotic segment of UPJO lesions, a significant reduction in the expression of PARs may inhibit the function of the SK3 channel, attenuating the role of PDGFRα^+^ cells in mediating smooth muscle relaxation, and resulting in ICCs predominantly mediating smooth muscle contraction. This persistent smooth muscle hypercontraction at the UPJ may ultimately lead to abnormalities in the distribution and function of SMCs (including hypertrophy/hyperplasia, atrophy/hypoplasia), consequently leading to abnormal peristaltic function in the UPJ and the development of UPJO.

This study aimed to examine the clinical implications of PARs on the pathogenesis of children with UPJO. All 16 patients in the UPJO group exhibited a preoperative SFU grade 4. Subsequently, the patients were further classified according to the Onen grading system (13). This system distinguishes SFU grade 4 into Onen grade 3 and Onen grade 4 based on whether the loss of renal parenchymal thickness on the affected side exceeds 1/2. Thereafter, the patients were categorized into three groups: the control group, the Onen grade 3 group, and the Onen grade 4 group. The RT-PCR analysis demonstrated a significantly lower PAR1 mRNA expression in the Onen grade 4 group compared to the controls. In addition, the statistical analysis demonstrated a significant disparity in the mRNA expression levels of PAR2 across the three groups. Specifically, the relative mRNA expression of PAR2 was significantly lower in the Onen grade 4 group compared to both the control group and the Onen grade 3 group. Furthermore, a significantly lower relative mRNA expression of PAR2 was observed in the Onen grade 3 group compared to the control group.

In summary, the present study suggests that low PAR2 expression may result in the downregulation of SK3 channel activity on the cytoplasmic membrane of downstream PDGFRα^+^ cells. Consequently, this downregulation leads to excessive contraction of SMCs and, may cause aberrant distribution and function of these cells in later stages. Therefore, a substantial decrease in PAR2 expression may indicate the presence of UPJO in patients. Given the structural and functional similarities between PAR1 and PAR2, a significant decrease in PAR1 expression implies a higher preoperative Onen grade in patients with UPJO.

One limitation of the study pertains to the utilization of the UPJ of patients diagnosed with nephroblastoma as a control group, which may potentially impact the outcomes. Given the challenge in procuring a sufficient number of normal UPJ tissues from children to establish a control group, we contend that nephroblastoma represents the primary, if not exclusive, option for obtaining unaffected UPJ samples for analysis. Additionally, animal and *in vitro* cell studies were not performed to thoroughly explore the underlying mechanism.

## Conclusions

Our study presents novel findings regarding the role of PARs in the pathogenesis of children with UPJO. PARs are widely distributed within the SIP syncytium and play an essential role in maintaining the equilibrium of smooth muscle cells' membrane potential through ICCs/ANO1 channels and PDGFRα^+^ cells/SK3 channels. Notably, the decreased expression of PAR1 suggests a higher preoperative Onen grade in UPJO patients. The downregulation of PAR2 at the UPJ may result in the loss of inhibitory neuromuscular transmission, thereby disrupting the rhythmic peristalsis at the UPJ. These conditions may lead to the failure of peristalsis at the UPJ, ultimately contributing to the development of congenital UPJO.

## Data Availability

The original contributions presented in the study are included in the article/Supplementary Material, further inquiries can be directed to the corresponding author.
